# Ectopic expression of Nav1.7 in spinal dorsal horn neurons induced by NGF contributes to neuropathic pain in a mouse spinal cord injury model

**DOI:** 10.3389/fnmol.2023.1091096

**Published:** 2023-03-03

**Authors:** Yan Fu, Liting Sun, Fengting Zhu, Wei Xia, Ting Wen, Ruilong Xia, Xin Yu, Dan Xu, Changgeng Peng

**Affiliations:** ^1^Key Laboratory of Freshwater Aquatic Genetic Resources, Ministry of Agriculture and Rural Affairs, National Pathogen Collection Center for Aquatic Animals, Shanghai Ocean University, Shanghai, China; ^2^The First Rehabilitation Hospital of Shanghai, Brain and Spinal Cord Innovation Research Center, School of Medicine, Advanced Institute of Translational Medicine, Tongji University, Shanghai, China; ^3^Pre-clinical College, Dali University, Dali, Yunnan, China

**Keywords:** Nav1.7, spinal cord injury, NGF, JUN, neuropathic pain, FOS

## Abstract

Neuropathic pain (NP) induced by spinal cord injury (SCI) often causes long-term disturbance for patients, but the mechanisms behind remains unclear. Here, our study showed SCI-induced ectopic expression of Nav1.7 in abundant neurons located in deep and superficial laminae layers of the spinal dorsal horn (SDH) and upregulation of Nav1.7 expression in dorsal root ganglion (DRG) neurons in mice. Pharmacologic studies demonstrated that the efficacy of the blood–brain-barrier (BBB) permeable Nav1.7 inhibitor GNE-0439 for attenuation of NP in SCI mice was significantly better than that of the BBB non-permeable Nav1.7 inhibitor PF-05089771. Moreover, more than 20% of Nav1.7-expressing SDH neurons in SCI mice were activated to express FOS when there were no external stimuli, suggesting that the ectopic expression of Nav1.7 made SDH neurons hypersensitive and Nav1.7-expressing SDH neurons participated in central sensitization and in spontaneous pain and/or walking-evoked mechanical pain. Further investigation showed that NGF, a strong activator of Nav1.7 expression, and its downstream JUN were upregulated after SCI in SDH neurons with similar distribution patterns and in DRG neurons too. In conclusion, our findings showed that the upregulation of Nav1.7 was induced by SCI in both SDH and DRG neurons through increased expression of NGF/JUN, and the inhibition of Nav1.7 in both peripheral and spinal neurons alleviated mechanical pain in SCI mice. These data suggest that BBB permeable Nav1.7 blockers might relieve NP in patients with SCI and that blocking the upregulation of Nav1.7 in the early stage of SCI *via* selective inhibition of the downstream signaling pathways of NGF or Nav1.7-targeted RNA drugs could be a strategy for therapy of SCI-induced NP.

## 1. Introduction

NP following SCI is a debilitating and distressing condition leading to sleep disturbances and depression (Burke et al., 2017; Widerström-Noga, 2017). The prevalence of NP following SCI is about 38%–70% (Werhagen et al., 2007; Burke et al., 2017; Kim et al., 2020), and the severe pain in SCI patients is primarily spontaneous due to central sensitization (Shiao and Lee-Kubli, 2018). NP induced by SCI is hard to manage because the complicated mechanisms behind it have not been fully uncovered yet.

Voltage-gated sodium channels (VGSCs) play critical roles in pain sensation and conduction, and are potential targets for pain relief. The first-generation unspecific VGSCs inhibitors lidocaine and mexiletine are local anesthetics used in clinics but have side effects in the brain and heart due to their inhibition of Nav1.1, Nav1.2, and Nav1.5 (Eijkelkamp et al., 2012). Therefore, the idea to develop second-generation sodium channel blockers with Nav-subtype selectivity was raised. Nav1.8 (SCN10A, also named PN3 and SNS), a member of the VGSC family, was first identified to be a Tetrodotoxin-resistant sodium channel and primarily expressed in the small neurons of rat DRG by Akopian et al. (1996) and Sangameswaran et al. (1996). Functional studies showed that Nav1.8 knockout mice have elevated mechanical pain thresholds to noxious pressure and also have deficits in inflammatory and visceral pain, but not in neuropathic pain (Akopian et al., 1999; Laird et al., 2002). Moreover, Nav1.8 gain-of-function mutations were found in patients with painful small-fiber neuropathy or with lower mechanical pain sensitivity (Faber et al., 2012; Duan et al., 2016; Han et al., 2018).

Nav1.7 (SCN9A, also named PN1), another member of VGSCs, was originally found in mice by Beckers et al. (1996) and Kozak and Sangameswaran (1996) and found to be principally expressed in peripheral neurons (Sangameswaran et al., 1997; Toledo-Aral et al., 1997). Lai et al. (2000) reported that knocking down the expression of Nav1.7 using *Scn9a* antisense in DRG neurons alleviated neuropathic pain. Following this, it was found that gain-of-function mutations of SCN9A caused inherited erythromelalgia, idiopathic small-fiber neuropathies, and spontaneous pain (Cummins et al., 2004; Yang et al., 2004; Faber et al., 2012; Xue et al., 2022). Knockout of *Scn9a* in DRG neurons of mice attenuated mechanical pain, inflammatory pain, and certain types of heat pain (Nassar et al., 2004; Minett et al., 2012), and the loss of function mutation of SCN9A leads to congenital insensitivity to pain in humans (Cox et al., 2006).

Given the strong clinical relevance of Nav1.7 and Nav1.8 in neuropathic pain, second-generation sodium channel blockers selectively targeting Nav1.7 or Nav1.8 have been developed since 2009, but none of them have yet achieved efficient effects in the attenuation of neuropathic pain in clinical trials. We previously found that Nav1.7 and Nav1.8 were upregulated in SDH neurons of peripheral nerve-injured mice and contributed to NP (Sun et al., 2021). It is unknown whether Nav1.7 and Nav1.8 is also ectopically expressed in SDH neurons to participate in NP following SCI. Here we show that SCI also activated ectopic expression of Nav1.7 which led to the sensitization of SDH neurons and contributed to NP.

## 2. Materials and methods

### 2.1. Animals

Adult (8–11 weeks) C57BL/6N mice(Vital River, Beijing)were fed with food and water *ad libitum* and housed five per cage, at 21°C, 50% humidity, on a 12 h light:12 h dark schedule in the standard animal facility in accordance with the guidelines of Tongji University. All animal work was conducted under ethical permission from the Tongji University ethical review panel.

### 2.2. Measurement of mechanical threshold

Before measuring the mechanical threshold, the mice were placed on a metal mesh and covered with transparent plexiglass, allowing them to acclimate for 30 min. Mechanical threshold was measured according to the previously described procedure (Sun et al., 2021). Briefly, the paw-withdrawal threshold of the ipsilateral hind paws of sham and SCI mice was measured with a set of calibrated monofilaments (von Frey hairs) in order of increasing forces from 0.008 g to 2 g; the force which caused paw withdrawal for three times during continuous stimulation was recorded as the paw-withdrawal threshold. Each monofilament was applied for a maximum of five times. The response ratio of paw withdrawal was the ratio of the number of times the animal withdrew the paw to the total number of measurements.

### 2.3. Surgery

**Figure 1:**
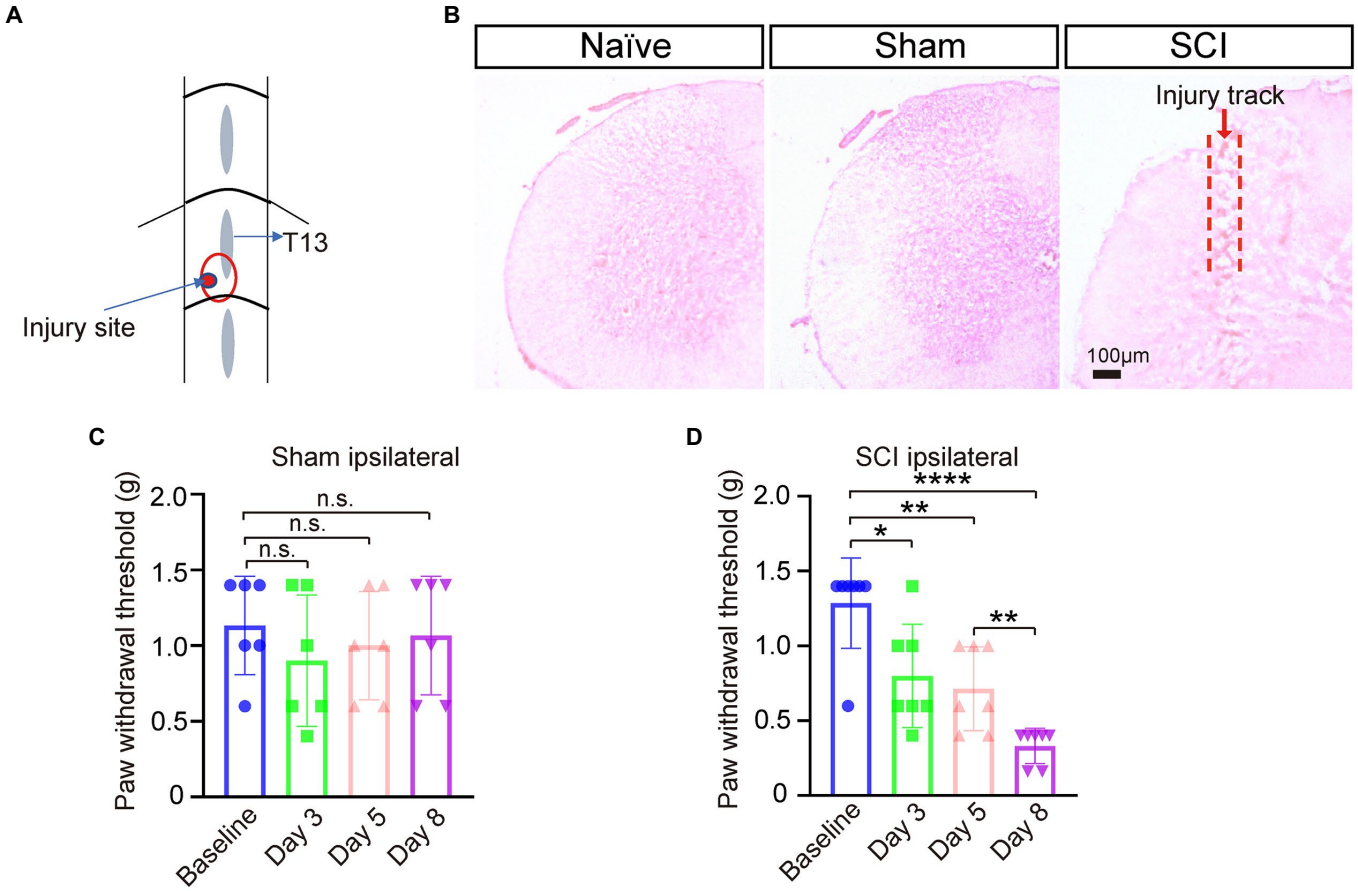
Neuropathic pain developed in SCI mice. **(A**) The SCI mouse model was generated by punching a hole in left L5 segment of spinal cord under vertebra thoracic segment 13 (T13) using a 26G needle. Injury site is marked with a red circle. **(B)** Hematoxylin and Eosin (H&E) staining of spinal cord sections showed that the injury track only presented in SCI mice, not in naive nor sham mice. **(C)** The paw-withdrawal threshold of the ipsilateral side of sham mice pre-surgery and three, five, and eight days post operation (DPO), *n* = 6. **(D)** The paw-withdrawal threshold of the ipsilateral side of SCI mice significantly dropped at 3 DPO and further decreased by 8 DPO. Baseline 1.3 ± 0.30 g; Day 3, 0.8 ± 0.35 g; Day 5, 0.7 ± 0.28 g; Day 8, 0.3 ± 0.12 g, *n* = 7. * *p* < 0.05, ** *p* < 0.01, **** *p* < 0.0001, unpaired *t-test* or one-way ANOVA. n.s. not significant. Scale bar = 100 μm.

Spinal cord injury (SCI) surgery was performed under isoflurane-induced anesthesia according to the protocol previously described (Sun et al., 2020) with a modification. Briefly, lumbar spinal segment L5 was exposed by laminectomy at vertebral level T13 (Figure 1A). Under a dissecting microscope, a small cut was made to the dura and arachnoid membrane followed by a defined lesion with a 26G needle (0.3 mm depth) unilaterally to the left dorsal horn, avoiding the dorsal root entry zone. Finally, skin was closed with 5–0 Nylon sutures. The surgical sham procedure was the same as SCI procedures except for injury of the dura, arachnoid membrane, and spinal cord. Mechanical pain threshold was measured at day 0 pre-surgery, and day 3, day 5, and day 8 after surgery. The mice with paw-withdrawal thresholds ≤0.4 g on day 8 after surgery were designated into the SCI group with NP and used for later experiment.

### 2.4. Drug treatments

From day 8 to day 11 after surgery, Nav1.7 channel blockers including PF-05089771 (Tocris, #5931, 2 mg/kg, and 4 mg/kg) and GNE-0439 (ProbeChem, # PC-62325, 10 μg/kg, 20 ug/kg, and 30 ug/kg) were administrated individually *via* IP injection into adult C57BL/6 SCI mice and mechanical pain threshold was tested at 30- and 60-min post drug administration. The voltage-gated calcium channel blocker Gabapentin (Sigma, #G154, 50 mg/kg) was administrated *via* IP injection into SCI mice and mechanical threshold was tested at 30- and 60-min post drug administration. Each drug test had a vehicle (saline or 20% DMSO) group and pre-treatment threshold was firstly measured each day.

### 2.5. Western blot

On day 12 after surgery, mice were anaesthetized and perfused using saline *via* left ventricle, and then L4-6 DRGs, L4-6 SDH, or L5 SDH from SCI mice, sham mice, and control mice were dissected and were immediately lysed with RIPA lysis buffer (Beyotime, China). The lysates were centrifuged at 4°C and 12,000 g for 10 min to pellet remaining cells and the cellular debris. About 20 μg DRG protein or 40 μg SDH protein from each animal were separated by SDS-PAGE in 10 and 6% (3:4) mixed cast gels and transferred onto PVDF membranes (Merck, USA). After 1h-blocking with 5% BSA in PBS, the membranes were incubated with primary antibody at 4 degree for overnight. Primary antibodies included: rabbit antibodies against Nav1.2 (Alomone labs, ASC-022, 1:100), NGF (Abcam, ab52918, 1:1,000), Phospho-c-JUN (Cell signaling technology, #9261, 1:1,000), and GAPDH (Proteintech, 60004-1-Ig, 1:3,000), and mouse antibodies against Nav1.7 (Abcam, ab85015, 1:800) and Nav1.8 (NeuroMab, 75–166, USA, 1:1,000). After washing three times in TBST, the membranes were further incubated with horseradish peroxidase (HRP)-conjugated horse anti-mouse secondary antibodies (Cell signaling technology, 7076S, 1:3,000), or HRP-conjugated goat anti-rabbit secondary antibodies (Cell signaling technology, 7074S, 1:3,000) for 1 h at room temperature. The proteins were visualized by chemiluminescent method using the ECL and detected using Western blot imaging machine (CLiNX, China). The intensity of each protein band was normalized to the intensity of GAPDH band to get the relative expression level of the interested protein.

### 2.6. Immunostaining

On day 12, mice were anaesthetized and perfused *via* left ventricle using saline, followed by 4% PFA, and then L4-6 DRGs, L4-6 SDH, or L5 SDH from SCI mice, sham mice, and control mice were dissected and were immersed in 4% PFA overnight. They were then soaked in 15% sucrose for 24 h and 30% sucrose for 24 h; afterwards, tissues were embedded in OCT. DRG and spinal cord tissues were sectioned in pieces of 14 μm thickness using a cryostat, and immunostaining was performed as previously described (Peng et al., 2017). The spinal cord sections and dorsal root ganglia sections were incubated overnight (2 days for Nav1.7 antibodies) at 4°C with primary antibodies, including rabbit antibodies against Nav1.7 (Proteintech Group, 20257-1-AP, 1:200), NGF (Abcam, ab52918, 1:300), and Phospho-c-JUN (Cell signaling technology, #9261, 1:300). The sections were further incubated with IgG Alexa Fluor 488 (Invitrogen, 1:1,000) and IgG Alexa Fluor 555 (Invitrogen, 1:1,000) secondary antibodies for 1.5 h at room temperature. According to the manufacturer’s instructions, we first used tyramide signal amplification (TSA) kits (PerkinElmer, NEL701A001KT, 1:50) to probe Nav1.7 (Proteintech Group, 20257-1-AP, 1:3,000) on the spinal cord sections, then incubated the rabbit antibodies against FOS (Abcam, ab190289, 1:1,000). The sections were further incubated with IgG Alexa Fluor 555 (Invitrogen, 1:1,000). Finally, the sections were counterstained with DAPI (Sigma, MBD0015, 1:10,000). Fluorescent images were captured by confocal laser scanning microscope (LSM780 or LSM880, Carl Zeiss, Oberkochen, Germany).

### 2.7. Statistical analysis

All quantitative data were presented as the mean ± standard deviation (SD). The data were analyzed using GraphPad Prism 9.0.0 software (GraphPad Software Inc., CA, USA), The data collected from von Frey test, protein level quantification, and immunofluorescence staining cell number were analyzed by unpaired *t*-test or one-way ANOVA, and the response ratios of paw withdrawal of SCI mice were analyzed by two-way ANOVA. A value of * *p* < 0.05 was considered as statistically significant.

## 3. Results

### 3.1. Upregulation of Nav1.7 in DRG of SCI mice

To investigate whether the expression levels of sodium channels changed in SDH and DRG after SCI, we generated SCI mouse models by making a restricted narrow lesion on the unilateral dorsal horn of lumbar segment 5 of the spinal cord while keeping the dorsal column and ventral horn intact (Figures 1A,B; Sun et al., 2020). Mechanical pain developed in the ipsilateral paws of SCI mice three days post operation (DPO), and the threshold of mechanical pain further lowered from three to eight DPO, but the paw-withdrawal threshold of sham mice did not significantly change after the sham operation (Figures 1C,D). These results indicated that the SCI model was successfully generated and NP was developed in these SCI mice.

**Figure 2:**
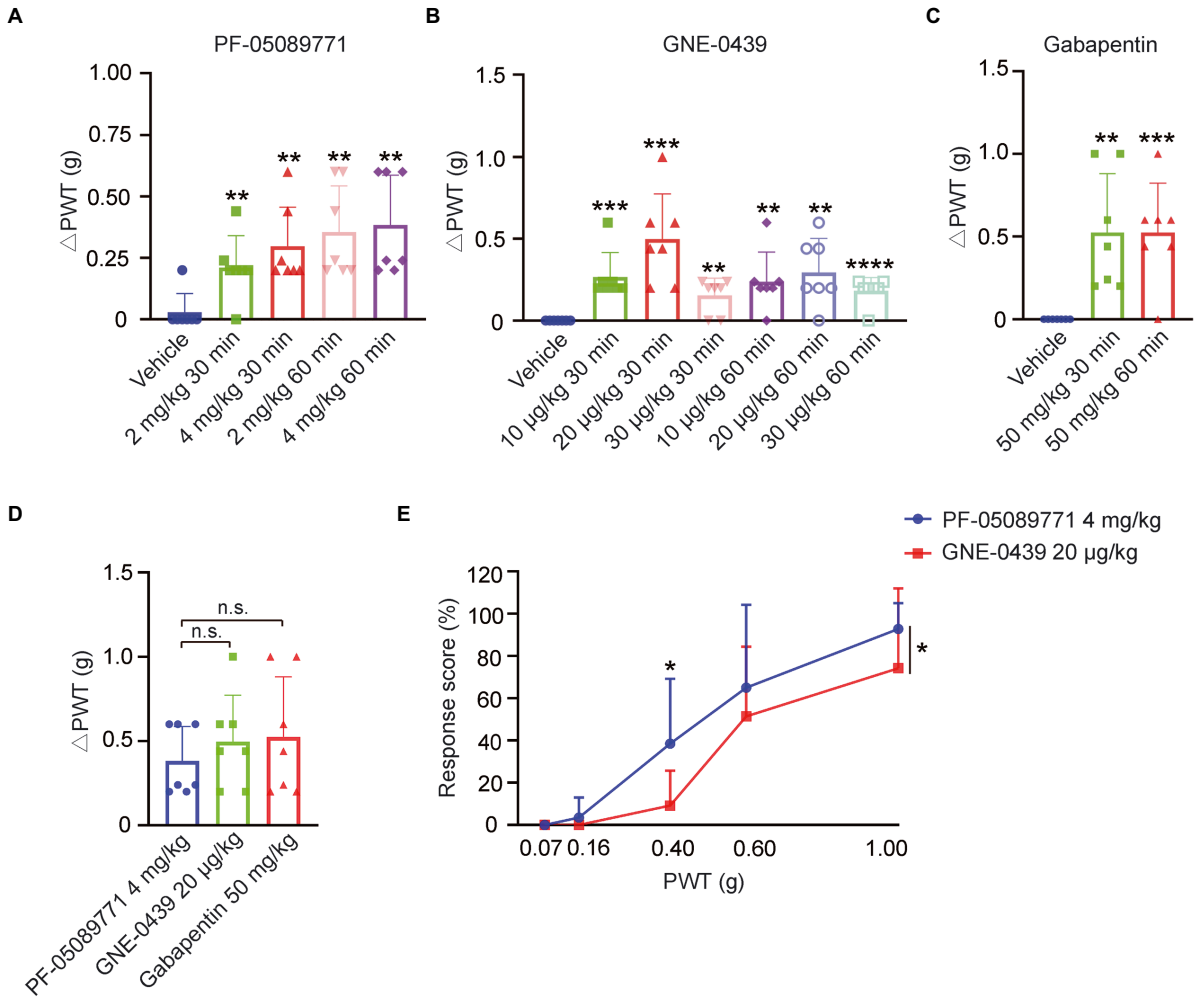
Expression of voltage-gated sodium channels in spinal dorsal horn and DRG of SCI mice at 12 DPO. **(A)** Western blot showed that the expression level of Nav1.2 was not significantly upregulated in SDH of SCI mice when compared to that in naive mice. **(B)** Western blot demonstrated that there was no significant difference in the expression level of Nav1.8 in SDH between SCI mice and naive mice. **(C)** The expression level of Nav1.7 in SDH of naive and SCI mice measured by Western-blot assay. **(D)** The expression level of Nav1.7 in DRG significantly increased 1.5-fold (1.5 ± 0.32) in SCI mice compared to that in naive mice. Data are shown as Mean ± SD, * *p* < 0.05, *n* = 4, unpaired *t-test*. n.s. not significant. Scale bar = 100 μm.

We then examined the expression level of Nav1.2 (SCN2A), Nav1.8 (SCN10A), and Nav1.7 (SCN9A) in SDH, and the result of the Western blot showed that none of the expression levels of Nav1.2, Nav1.8, or Nav1.7 in L4-6 segment SDH of SCI mice was significantly changed when compared to that in naive mice (Figures 2A–C, uncropped image of membrane showed in Supplementary Figure S1). However, these results showed an upregulation trend of Nav1.7 (1.1 ± 0.18) and Nav1.2 (1.1 ± 0.39) in SDH of the SCI mice (Figures 2A–C). It is possible that spinal cord injuries might affect the expression of Nav1.7 and Nav1.2 in DRG, so we detected the expression levels of Nav1.7 and Nav1.2 in DRG using Western blot. The results showed that Nav1.7 was significantly upregulated 1.5-fold in DRG of SCI mice when compared to that in naive mice (Figure 2D, uncropped image of membrane showed in Supplementary Figure S1), but the expression of Nav1.2 was undetectable in the DRG of both SCI and naive mice (data not shown).

### 3.2. Ectopic expression of Nav1.7 in SDH neurons in SCI mice

**Figure 3:**
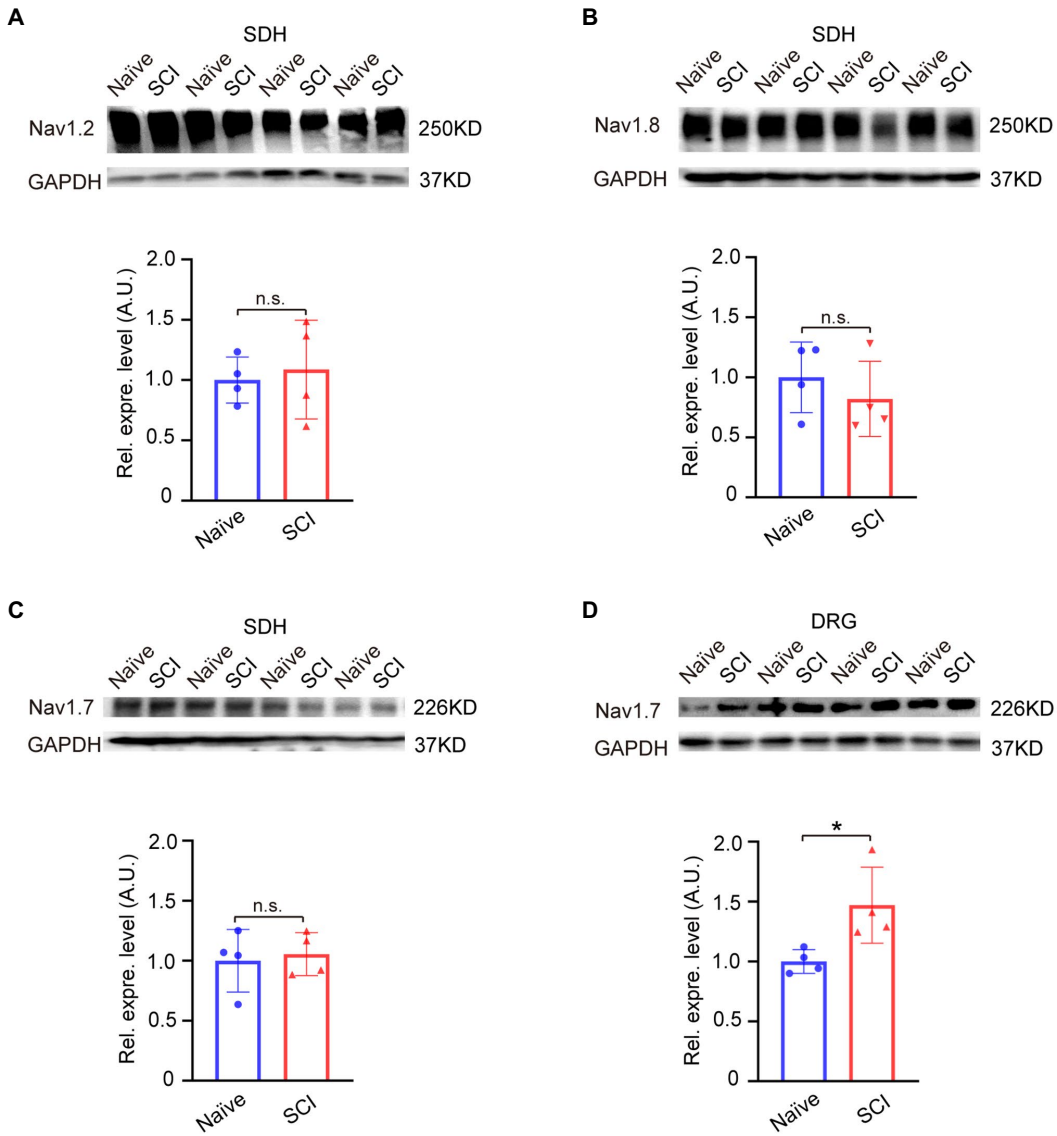
Upregulation of Nav1.7 in DRG and SDH of SCI mice. **(A)** The expression level of Nav1.7 in DRG and SDH of naive, sham, and SCI mice was measured by Western blot. **(B)** Quantitative analysis of duplicate Western-blot membranes showed that compared to naive mice, the expression level of Nav1.7 increased significantly in DRG of SCI mice (1.3 ± 0.10), and an increasing trend in expression level of Nav1.7 was also observed in sham mice (1.1 ± 0.19). The expression level of Nav1.7 in SDH of SCI and sham mice was 2.2-fold and 1.5-fold higher than that in naive mice, respectively, (2.2 ± 1.50, 1.5 ± 1.03). **(C)** Immunostaining for Nav1.7 (Green) on spinal section derived from naive, sham, and SCI mice. Arrows point to Nav1.7 positive neurons, DAPI is counterstaining for nuclei. Inset is the high magnification view of the boxed area. **(D)** The number of neurons expressing Nav1.7 in laminae I-VI of naive, sham, and SCI mice. # represents the comparison between SCI and sham，* represents the comparison between naïve and SCI or sham. **(E)** Immunostaining for Nav1.7 (Green) on DRG section derived from naive, sham, and SCI mice demonstrated the upregulation of Nav1.7 in DRG neurons in SCI and sham mice when compared to that in naive mice. Inset is the high magnification view of the boxed area. Data are shown as Mean ± SD, *n* = 3. * *p* < 0.05, ** *p* < 0.01, *** *p* < 0.001, ^##^ *p* < 0.01, ^###^ *p* < 0.001, unpaired *t-test* or one way ANOVA. n.s. not significant. Scale bar = 100 μm.

We previously found that sham operations (cutting open the skin of the leg and destroying the peripheral nerve of DRG neurons in the skin) altered the expression *Scn9a* mRNA in SDH. To decipher the contributions of spinal injuries and sham operations, which include peripheral injuries in the back skin and drilling a hole in vertebrate to NP, we employed sham group mice and found that there was an increase of Nav1.7 expression in L4-6 DRG of sham mice (1.1-fold), but no statistical significance when compared to that in naive mice; the expression of Nav1.7 in L4-6 DRG was significantly upregulated 1.3-fold more in SCI mice than that in naive mice (Figures 3A,B, uncropped image of membrane showed in Supplementary Figure S2). These data indicated that spinal cord injuries indeed contributed to the upregulation of Nav1.7 in DRG although the sham operation of SCI surgery also mildly enhanced the expression of Nav1.7 in L4-6 DRG.

As the damaged area of the spinal cord was small due to only one minor lesion being made in our model, in order to measure the change of gene expression in injured tissue we then dissected injury-site-contained L5 segment SDHs only and excluded the L4 and L6 segments of SDH which were further from the injury site. The Western-blot results showed that the expression level of Nav1.7 was increased 1.5-fold and 2.2-fold in SDH of sham and SCI mice, respectively, although it was not statistically significant (Figures 3A,B). In order to understand if SDH neurons in SCI mice gained expression of Nav1.7, we performed immunostaining for Nav1.7 and found that the number of Nav1.7-expressing neurons significantly increased 2.9-fold and 12.9-fold in SDH of sham (15 ± 3.1 cells per section) and SCI (64 ± 4.9 cells per section) mice, respectively, when compared to that in naive mice (5 ± 1.7 cells per section) (Figures 3C,D). The increased Nav1.7-expressing neurons were distributed from laminae I-VI in SCI mice (Figure 3D). Immunostaining on DRG sections also showed upregulation of Nav1.7 in DRG neurons of SCI mice and sham mice when compared to that in naive mice (Figure 3E).

### 3.3. Blockers of Nav1.7 alleviated mechanic pain of SCI mice

**Figure 4:**
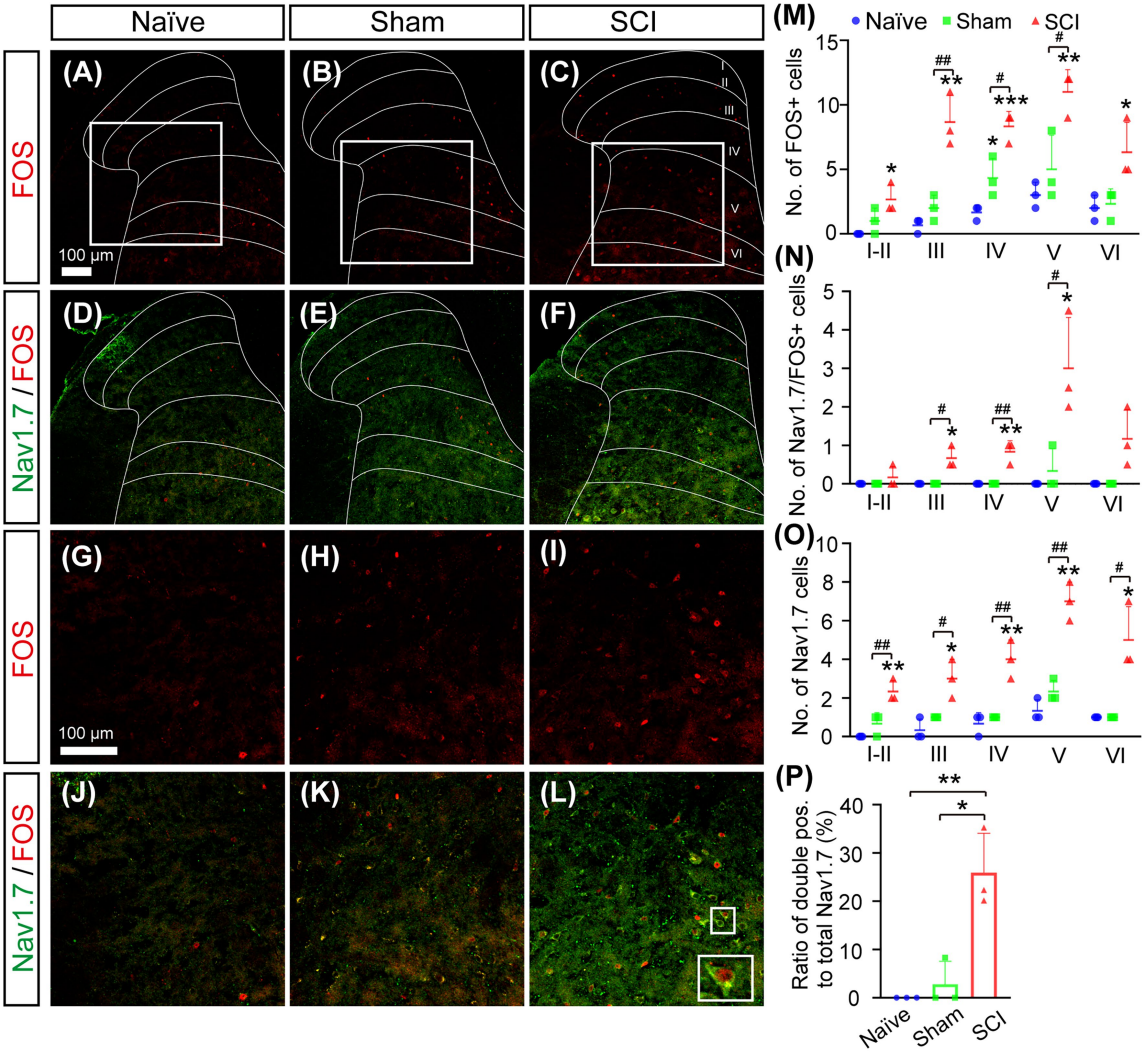
Blockers of Nav1.7-alleviated mechanical pain in SCI mice. **(A)** Nav1.7 blocker PF-05089771 significantly relieved mechanical pain of SCI mice at doses of both 2 mg/kg and 4 mg/kg (Vehicle 0.03 ± 0.08 g, 2 mg/kg 30 min 0.2 ± 0.13 g, 4 mg/kg 30 min 0.3 ± 0.16 g, 2 mg/kg 60 min 0.4 ± 0.19 g, and 4 mg/kg 60 min 0.4 ± 0.20) g. △PWT = post-drug PWT - pre-drug PWT. **(B)** Nav1.7 blocker GNE-0439 significantly alleviated mechanical pain of SCI mice at doses from 10 μg/kg to 30 μg/kg (10 μg/kg 30 min 0.3 ± 0.15 g, 20 μg/kg 30 min 0.5 ± 0.28 g, 30 μg/kg 30 min 0.2 ± 0.11 g, 10 μg/kg 60 min 0.2 ± 0.18 g, 20 μg/kg 60 min 0.3 ± 0.20 g, and 30 μg/kg 60 min 0.2 ± 0.08 g). **(C)** Gabapentin significantly reduced mechanical pain of SCI mice at dose of 50 mg/kg (30 min 0.5 ± 0.36 g and 60 min 0.5 ± 0.30 g). **(D)** The efficacy of GNE-0439 to relieve mechanical pain was equivalent to Gabapentin, and the efficacy of GNE-0439 and Gabapentin was slightly better than PF-05089771, but not statistically significant (PF-05089771 0.4 ± 0.20 g, GNE-0439 0.5 ± 0.28 g, and Gabapentin 0.5 ± 0.36 g). **(E)** Response ratio of paw withdrawal of SCI mice administrated neither PF-05089771 (blue line) or GNE-0439 (red line) to stimulus from 0.07 g Von Frey monofilament to 1.0 g Von Frey monofilament. Data are shown as Mean ± SD, *n* = 7. *p < 0.05, ** *p* < 0.01, *** *p* < 0.001, **** *p* < 0.0001, unpaired *t-test* or one-way ANOVA, or two-way ANOVA. n.s. not significant.

In order to confirm if the upregulation of Nav1.7 in DRG contributed to NP induced by SCI, we employed the blood–brain-barrier (BBB) non-permeable blocker of Nav1.7, PF-05089771(McDonnell et al., 2018). Intraperitoneal injection of 2 mg/kg PF-05089771 significantly alleviated mechanical pain when compared to vehicle (Figure 4A), and 4 mg/kg of PF-05089771 did not increase the efficacy of pain relief in SCI mice (Figure 4A). These data indicated that the upregulation of Nav1.7 in DRG indeed participated in mechanical allodynia. To investigate if the ectopic expression of Nav1.7 in SDH neurons contributed to NP in SCI mice, we employed another Nav1.7 blocker GNE-0439 (Chernov-Rogan et al., 2018) which is able to penetrate BBB (data not shown). GNE-0439 also attenuated mechanical pain in SCI mice significantly at a dose range from 10 to 30 μg/kg, with maximal efficacy at a dose of 20 μg/kg, but the effects of GNE-0439 declined 30 to 60 min post drug administration (Figure 4B). Intraperitoneal injection of 50 mg/kg Gabapentin also significantly relieved mechanical pain in SCI mice (Figure 4C). However, Gabapentin at this dose caused sedation and movement disability in one out of seven tested mice (data not shown). Although there was not a statistically significant difference, the maximal efficacy of GNE-0439 and Gabapentin in the SCI mice was slightly better than that of PF-05089771 (PF-05089771 0.4 ± 0.20 g, GNE-0439 0.5 ± 0.28 g, Gabapentin 0.5 ± 0.36 g) (Figure 4D). GNE-0439 had equivalent efficacy of pain relief as Gabapentin, but no sedation side effect. We further compared the response ratio of SCI mice to mechanical pressing stimulus from 0.07 g Von Frey to 1.0 g Von Frey after administration of Nav1.7 blockers, and found that GNE-0439 showed better effects to alleviate mechanical pain than PF-05089771 did (Figure 4E). These data suggested that the upregulation of Nav1.7 in DRG and SDH may contribute to NP in SCI mice.

### 3.4. Nav1.7-expressed SDH neurons involved in NP

**Figure 5:**
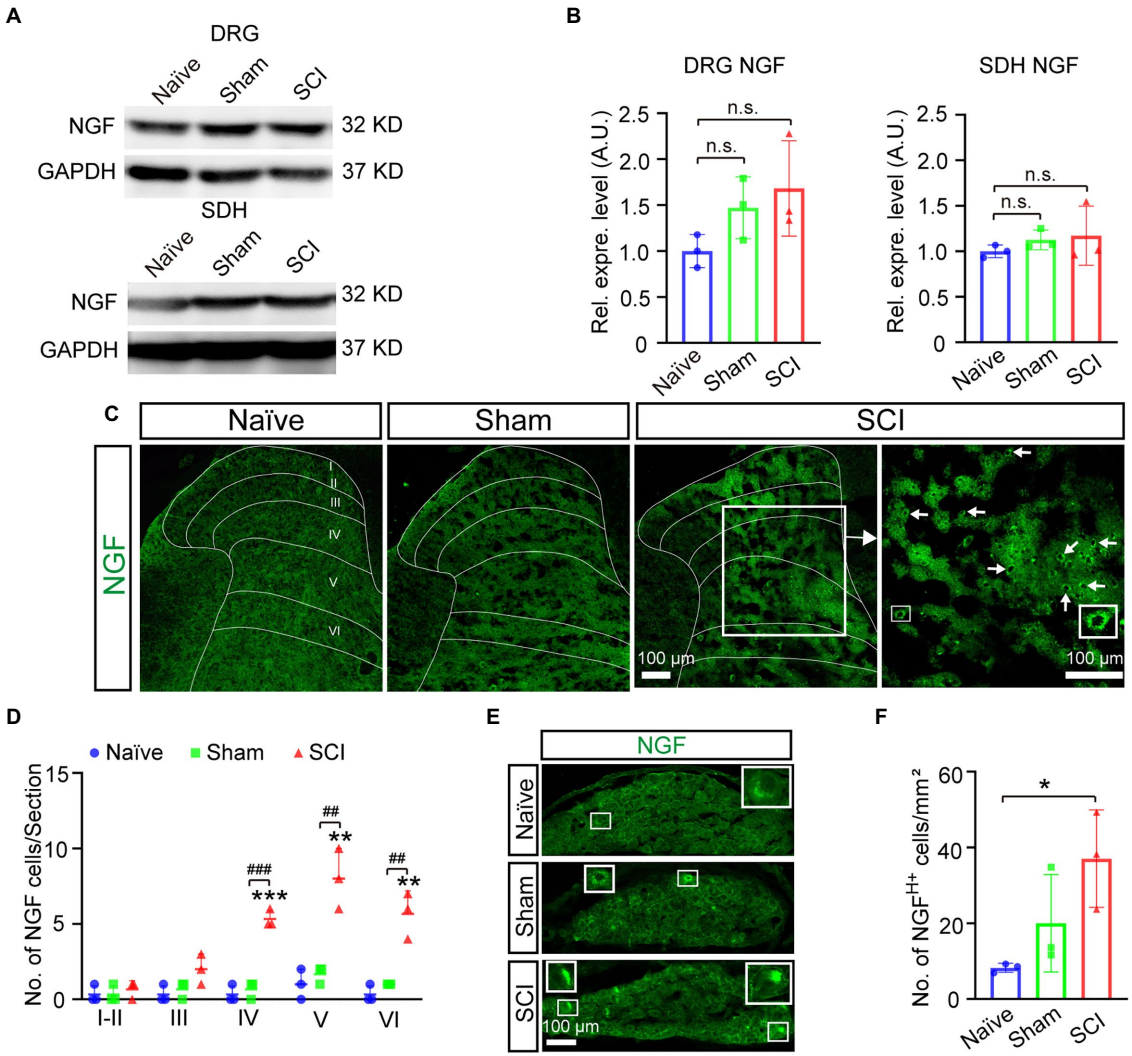
Activation of Nav1.7^+^ SDH neurons in SCI mice. **(A–L)** Double immunostaining for FOS (Red) and Nav1.7 (Green) on spinal section derived from naive **(A, D, G, J)**, sham **(B, E, H, K)**, and SCI **(C, F, I, L)** mice showed more FOS-positive neurons and more FOS/Nav1.7 double-positive neurons in deep laminae layers of SCI mice. Inset is the high magnification view of the boxed area. Scale bar 100 μm. **(M–O)** The number of FOS^+^ **(M)**, Nav1.7^+^/FOS^+^ **(N)**, and Nav1.7^+^ **(O)** SDH neurons in laminae I–VI of home-caged naive mice, sham, and SCI mice. The symbol # represents the comparison between SCI and sham, and the symbol * represents the comparison between naive and SCI or sham. **(P)** The ratio of Nav1.7^+^/FOS^+^ SDH neurons to total Nav1.7^+^ SDH neurons in naive (0%), sham (3 ± 4.8%), and SCI (26 ± 8.2%) mice. Data are shown as Mean ± SD, *n* = 3. ^#^ *p* < 0.05, ^##^ *p* < 0.01,*p < 0.05, ** *p* < 0.01, ***p < 0.001, unpaired *t*-test or one way ANOVA. Scale bar = 100 μm.

Nav1.7 plays an important role in determination of action potential threshold (Bennett et al., 2019), and we previously found that mild mechanical pressing caused mechanical allodynia and activated FOS expression in SDH neurons with ectopic expression of Nav1.7 in *Gad2^CreERT2/+^*; *miR-96^flox/flox^* mice which had ablation of *miR-96* in GAD2 neurons in SDH, but not in DRG (Sun et al., 2021). To investigate if SDH neurons with ectopic expression of Nav1.7 in SCI mice participated in pain conductions, we performed double immunostaining for Nav1.7 and FOS, which is a marker indicating the activation of neurons. The results showed the number of activated neurons with FOS expression increased significantly more in SCI mice than that in sham and naive mice (Figures 5A–M). Moreover, the number of Nav1.7/FOS double-positive neurons was also significantly elevated in laminae III–V of SCI mice (Figures 5D–F,N). Sham mice and naive mice had only a few and no neurons expressing both Nav1.7 and FOS in SDH, respectively. Further analysis showed that more than one quarter (26 ± 8.2%) of Nav1.7-expressing SDH neurons were activated in SCI mice which had mechanical stimuli mainly from walking in their home cages (Figures 5O,P). These data together suggested that SCI mice had mechanical pain during walking in their home cages and that ectopic expression of Nav1.7 made neurons in deep laminae layers hyperactive and participatory in the conduction of mechanical pain.

### 3.5. SCI induced the elevation of NGF and phosphorylated-JUN in DRG and SDH mice

**Figure 6:**
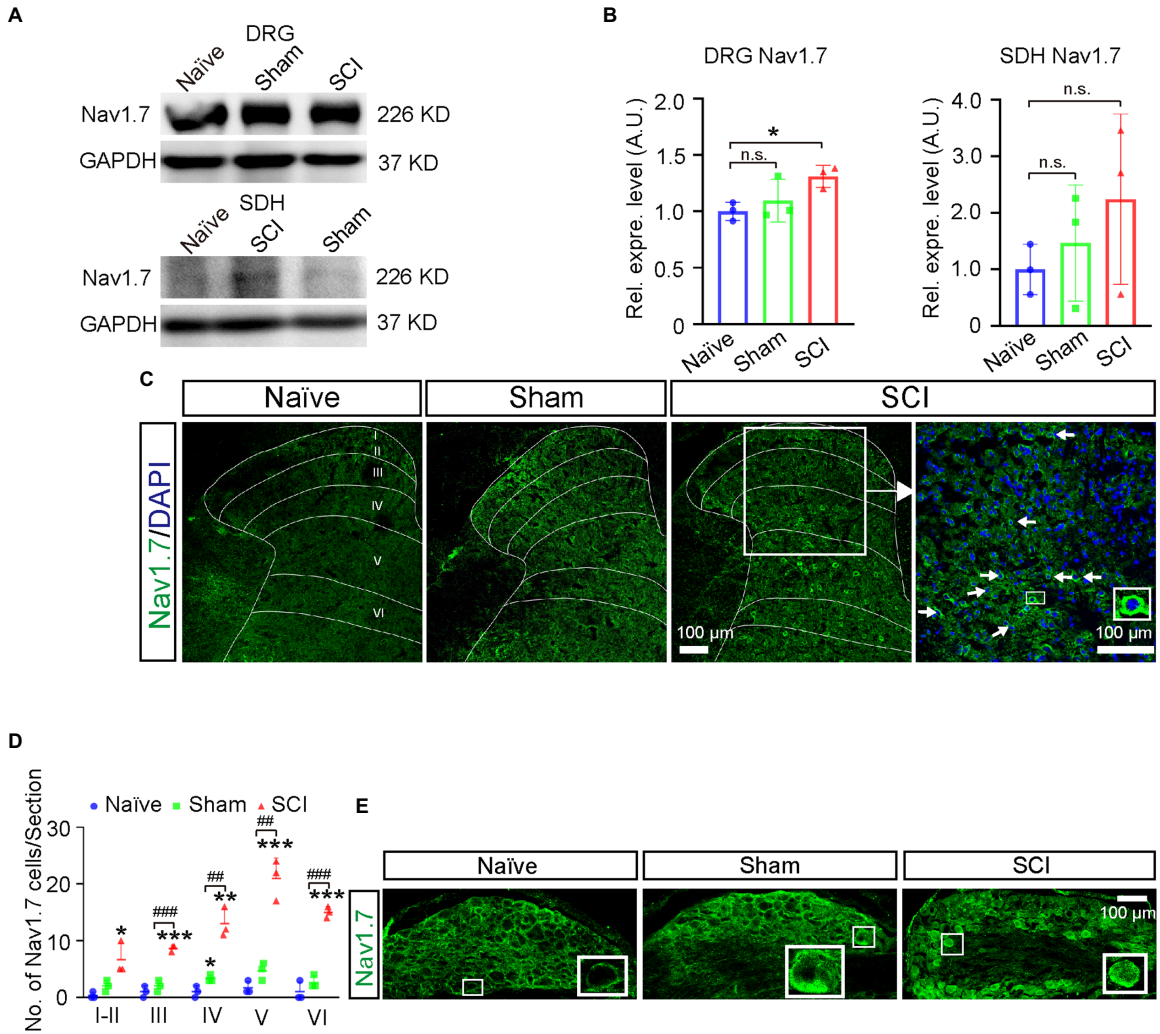
Induced expression of NGF in DRG and SDH of mice after SCI. **(A)** The expression level of NGF in DRG and SDH of naive, sham, and SCI mice was measured by Western blot. **(B)** Quantitative analysis of duplicate Western-blot membranes showed that there was an increase trend in the expression level of NGF in DRG and SDH in SCI and sham mice when compared to naive mice (sham DRG 1.5 ± 0.34, SCI DRG 1.7 ± 0.52; sham SDH 1.1 ± 0.11, SCI SDH 1.2 ± 0.32). **(C)** Immunostaining for NGF (Green) on spinal cord section derived from naive, sham, and SCI mice. Arrows point to NGF positive neurons. Inset is the high magnification view of the boxed area. **(D)** The number of neurons expressing NGF in laminae I-VI of naive, sham, and SCI. # represents the comparison between SCI and sham, * represents the comparison between naive and SCI or sham. **(E, F)** Immunostaining for NGF (Green) on DRG section derived from naive, sham, and SCI mice **(E)** and the number of neurons expressing high levels of NGF in DRG neurons in naive, sham, and SCI mice. Inset is the high magnification view of the boxed area. Data are shown as Mean ± SD, *n* = 3. ## *p* < 0.01, ###*p* < 0.001, *p < 0.05, **p < 0.01, ***p < 0.001,unpaired *t-test* or one way ANOVA. n.s. not significant. Scale bar = 100 μm.

**Figure 7:**
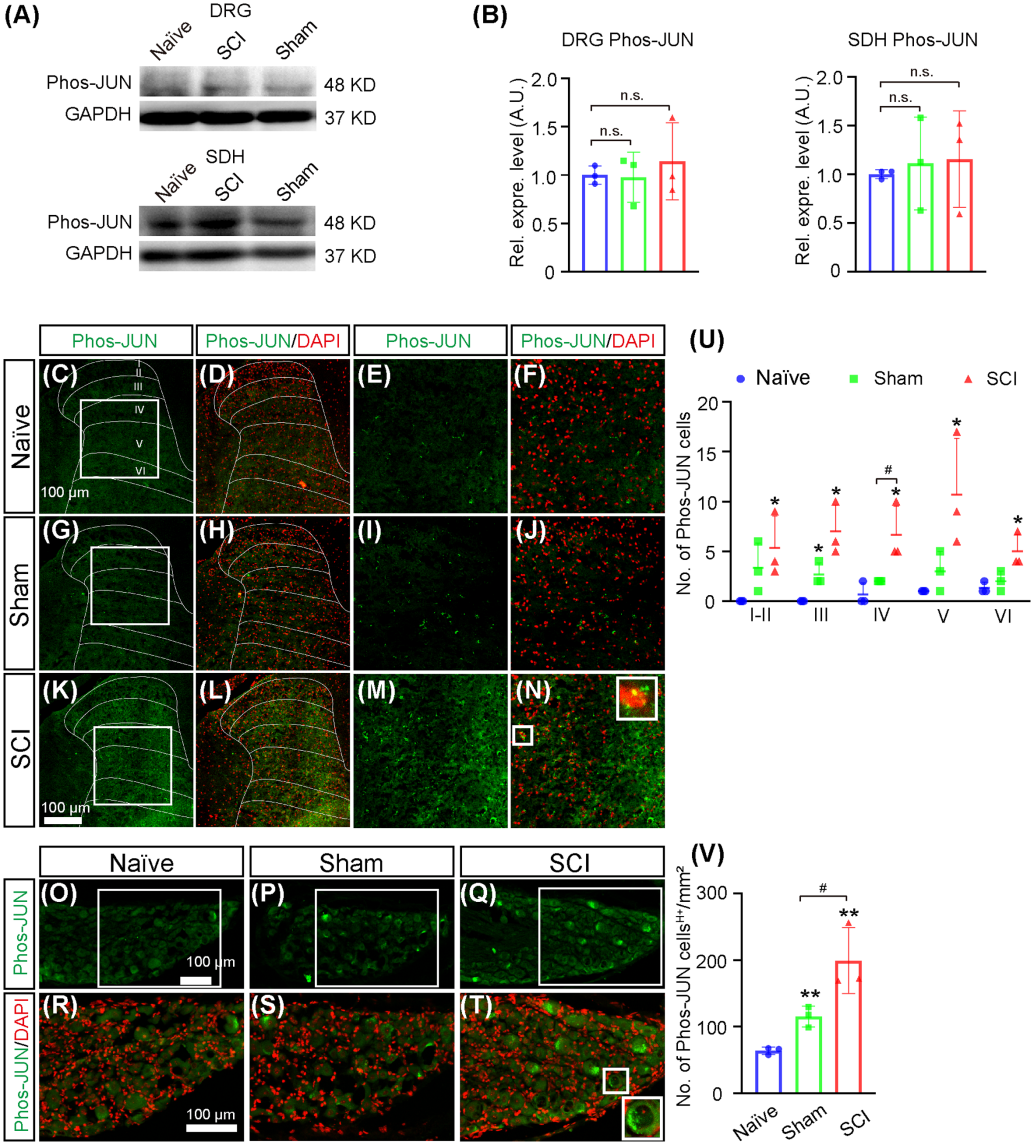
Increase of phosphorylated JUN in SDH and DRG of SCI mice. **(A)**. The expression level of phosphorylated JUN in DRG and SDH of naive, sham, and SCI mice was measured by Western blot. **(B)** Quantitative analysis of the expression of phosphorylated JUN in DRG and SDH in naive, sham, and SCI (sham DRG 1.0 ± 0.26, SCI DRG 1.1 ± 0.40; sham SDH 1.1 ± 0. 48, SCI SDH 1.2 ± 0.50). **(C–N)** Immunostaining for phosphorylated JUN (green) and DAPI (red) staining on spinal cord sections derived from naive **(C–F)**, sham **(G–J)**, and SCI mice **(K–N)**. **(E-F, I–J)** and **(M–N)** are the high magnification views of the boxed area in **(C,D**), **(G,H)**, and **(K, L)**, respectively. The inset (right) in **(N)**, which is the high magnification view of the boxed area in **(N)** (left), shows phosphorylated JUN in the nucleus. **(O)** The number of neurons expressing phosphorylated JUN in laminae I–VI of naive, sham and SCI mice. **(P–U)** Immunostaining for phosphorylated JUN (green) and DAPI (red) staining on DRG sections derived from naive **(P, S)**, sham **(Q, T)**, and SCI **(R, U)** mice. **(S–U)** are the high magnification views of the boxed area in **(P–R)**, respectively. The inset (right) in **(U)**, which is the high magnification view of the boxed area in (**U**, left) shows phosphorylated JUN in the nucleus. **(V)** The number of neurons expressing phosphorylated JUN in DRG of naive, sham, and SCI mice. # represents the comparison between SCI and sham. * represents the comparison between naive and SCI or sham. Data are shown as Mean ± SD, *n* = 3. #*p* < 0.05, *p < 0.05, **p < 0.01, unpaired t-test or one-way ANOVA. n.s. not significant. Scale bar = 100 μm.

It was reported that the expression of Nav1.7 in DRG could be induced by nerve growth factor (NGF) (Toledo-Aral et al., 1997; Liu et al., 2021), so we examined whether the expression of NGF was upregulated after SCI. The results of the Western blot showed that the expression level of NGF increased 1.7-fold and 1.2-fold in DRG and SDH, respectively, in SCI mice when compared to that in naive mice (Figures 6A,B, uncropped image of membrane showed in Supplementary Figure S3), although it was not statistically significant. We then detected the expression of NGF on a cellular level by immunostaining and found that the number of NGF-expressing SDH neurons significantly increased in laminae IV–VI of SCI mice when compared to those in both sham and naive mice (Figures 6C,D). The immunostaining also demonstrated that the number of neurons expressing high levels of NGF (NGF^H+^) was upregulated in L4-6 DRG of SCI and sham mice when compared to that in naive mice (Figures 6E,F), which was in line with previous reports that the expression of NGF in DRG was induced by injuries of the skin and muscle (Liu et al., 2021). We then asked how NGF activated transcription of *Scn9a*. It is known that NGF can activate MAPK/ERK signaling pathways in DRG neurons (Obata et al., 2004) and that MAPK/ERK activates JUN (Raitano et al., 1995). We found that there are predicted binding sites of JUN and FOS on the promoter of *Scn9a*^1^, so it is possible that NGF induces the upregulation of Nav1.7 through MAPK/ERK/JUN/FOS. Therefore, we examined the expression of phosphorylated JUN by Western blot and immunostaining. The result of Western blot showed that the expression levels of phosphorylated JUN was not significantly increased in DRG and SDH of SCI mice when compared to that in naive mice (Figures 7A,B, and uncropped image of membrane showed in Supplementary Figure S4). However, the number of phosphorylated JUN-expressing SDH neurons significantly increased in laminae I–VI of SCI mice when compared to naive mice, and SCI mice had more phosphorylated JUN-expressing SDH neurons on laminae IV than sham mice (Figures 7C–O). The immunostaining also demonstrated that the number of neurons expressing high levels of phosphorylated JUN (Phos-JUN ^H+^) was upregulated in L4-6 DRG of SCI mice when compared to that in naive and sham mice (Figures 7P–V). Moreover, phosphorylated JUN was found in the nucleus of DRG and SDH neurons of SCI mice (Figures 7N,U). These data suggest that NGF induced Nav1.7 expression *via* transcription factor JUN in both SDH and DRG after spinal cord injury.

## 4. Discussion

**Figure 8:**
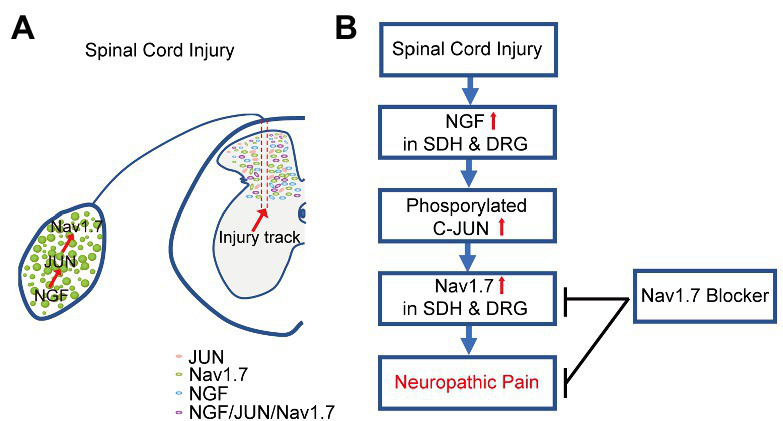
Schematic view of mechanism underlying SCI-induced NP. **(A)**. SCI induced the upregulation of NGF, and consequently increased phosphorylated JUN and the upregulation of Nav1.7 in SDH and DRG neurons of mice. **(B)**. Nav1.7 selective blockers attenuated SCI-induced NP in mice through inhibiting the activity of Nav1.7 in both the spinal cord and DRG.

Voltage-gated sodium channel Nav1.7 plays a vital role in physiological and pathological pain. Here, we demonstrated that SCI induced upregulation of NGF and JUN in both SDH and DRG neurons, subsequently increased the expression of Nav1.7 in SDH and DRG neurons to contribute to NP, and Nav1.7 selective blockers significantly attenuated NP in SCI mice (Figure 8).

Under physiological conditions, Nav1.7 is primary expressed in the peripheral nerve system (Toledo-Aral et al., 1997); here, we reported that Nav1.7 was induced to be expressed ectopically in SDH neurons located in both the superficial and deep layers surrounding the damaged spinal area in SCI mice (Figures 3C,D). It is known that neurons in deep layers (laminae III–VI) may be involved in pain conduction (Yu et al., 2011; Peng et al., 2017; Zain and Bonin, 2019), and the ectopic expression of Nav1.7 leads to hypersensitivity and hyperexcitability of SDH neurons in deep (III–V) and superficial layers (I–II) (Sun et al., 2021). The majority of Nav1.7-expressing neurons are located in laminae III–VI and more than 20% of these Nav1.7-expressing neurons were activated to express FOS in the SCI mice which had mechanical stimuli mainly from walking. This suggested that ectopic expression of Nav1.7 in laminae III–VI neurons participated in the conduction of mechanical and/or spontaneous pain. These data and our previous findings that peripheral nerve injury and knockout of *miR-96* led to ectopic expression of Nav1.7 in SDH neurons (Sun et al., 2021) together suggested that Nav1.7 was also expressed in SDH neurons and contributed to central sensitization and NP under pathological conditions, such as peripheral and spinal neuropathy.

Nav1.7 blockers were able to attenuate mechanical pain in the SCI mice, and GNE-0439 obtained an efficacy of pain relief equivalent to Gabapentin, but without the side effects. Moreover, in line with the increase of Nav1.7 expression in SDH neurons, BBB-permeable Nav1.7 blocker GNE-0439 relieved mechanical pain better than BBB non-permeable Nav1.7 blocker PF-05089771 (Figures 4D,E). However, the efficacy of GNE-0439 in SCI mice quickly dropped from 30 min after drug administration to 60 min after drug administration, suggesting that the half-life of GNE-0439 in mouse is short and its efficacy could be improved by increasing its metabolic stability and/or using a sustained-release tablet form. These pharmacological data suggest that BBB-permeable Nav1.7 blocker might efficiently attenuate NP in patients with SCI.

It was previously reported that primary DRG neuron culture treated with NGF upregulated the expression of Nav1.7 and increased the distribution of Nav1.7 in axon growth cone which was believed to play an important role for axon projection (Toledo-Aral et al., 1997); it is also known that SCI induced the expression of NGF mRNA and protein in both spinal cord and DRG neurons (Bakhit et al., 1991; Krenz and Weaver, 2000; Brown et al., 2004, 2007), so upregulation of NGF in DRG neurons found in the SCI mice was possible to help DRG neurons to regenerate axon projection through axon growth cone-located Nav1.7. The distribution patterns of Nav1.7-expressing SDH neurons (laminae I-VI) and NGF-expressing SDH neurons (majority in laminae IV-VI) overlapped, surrounding the damage area and with the majority of them located in deep layers (Laminae III–VI) (Figures 3C, 6C); this suggested that NGF was autocrine and/or paracrine to activate the expression of Nav1.7 in SDH neurons. Therefore, NGF induced by injury played contradictory roles in SCI. On one hand, it could promote the repairment of the spinal cord and survival of both spinal neurons and DRG neurons (Rich et al., 1989; Kim et al., 1996; Ljungberg et al., 1999); on the other hand, NGF caused central sensitization and peripheral hyperexcitability *via* increasing expression of Nav1.7 in both SDH and DRG neurons (Figure 3). Anti-NGF treatment or blocking NGF signaling pathways could reduce SCI-induced NP and osteoarthritic pain in animal models (Christensen and Hulsebosch, 1997; Krenz et al., 1999; Gwak et al., 2003; Hirose et al., 2016; Eitner et al., 2017), but in the meanwhile could also delay the reparation of the spinal cord. Our data showed that transcription factor JUN, downstream of NGF signaling pathways (Yu et al., 2011; Zain and Bonin, 2019) was increased in laminae I-VI of SCI mice in a similar pattern as Nav1.7 (Figure 7), and we found that there are predicted binding sites of JUN and FOS on the promoter of *Scn9a*^2^; together, these suggest that NGF induces upregulation of Nav1.7 through MAPK/ERK/JUN. One choice to eliminate the negative role of NGF is to block its activation of Nav1.7, which could be approached by inhibiting the downstream MAPK signaling pathways, but retaining the downstream PKC and NF-KB signaling pathways which promote survival, or could be approached by Nav1.7-targeted siRNAs (Lai et al., 2000) or miRNAs, such as miR-96 (Sun et al., 2021).

In conclusion, our data demonstrated that the upregulation of Nav1.7 was induced by SCI in both SDH and DRG neurons through increased expression of NGF and its downstream transcription factor JUN, and the inhibition of Nav1.7 in both peripheral and spinal neurons was better than the inhibition of peripheral Nav1.7 only in the alleviation of mechanical pain in SCI mice. These data suggest that BBB permeable Nav1.7 blocker might relieve NP in patients with SCI.

## Data availability statement

The original contributions presented in the study are included in the article/Supplementary material, further inquiries can be directed to the corresponding authors.

## Ethics statement

The animal study was reviewed and approved by Tongji University ethical review panel.

## Author contributions

CP: conceptualization and writing – original draft. YF, LS, FZ, WX, TW, and RL: methodology. YF and CP: investigation and visualization. CP and LS: funding acquisition. DX and CP: project administration and supervision. LS, XY, and DX: writing – review and editing. All authors contributed to the article and approved the submitted version.

## Funding

Changgeng Peng is supported by the National Natural Science Foundation of China (32070977, 51971236, 31871063) and National Major Science and Technology Projects of China (2018ZX09733001-006-005). Liting Sun is supported by the National Natural Science Foundation of China (82101320).

## Conflict of interest

The authors declare that the research was conducted in the absence of any commercial or financial relationships that could be construed as a potential conflict of interest.

## Publisher’s note

All claims expressed in this article are solely those of the authors and do not necessarily represent those of their affiliated organizations, or those of the publisher, the editors and the reviewers. Any product that may be evaluated in this article, or claim that may be made by its manufacturer, is not guaranteed or endorsed by the publisher.

## Footnotes

^1^ www.genecards.org

^2^ www.genecards.org

## References

[ref1] Akopian A. N. Sivilotti L. Wood J. N. (1996). A tetrodotoxin-resistant voltage-gated sodium channel expressed by sensory neurons. Nature 379, 257–262. doi: 10.1038/379257a0 8538791

[ref2] Akopian A. N. Souslova V. England S. Okuse K. Ogata N. Ure J.. (1999). The tetrodotoxin-resistant sodium channel SNS has a specialized function in pain pathways. Nat. Neurosci. 2, 541–548. doi: 10.1038/9195, PMID: 10448219

[ref3] Bakhit C. Armanini M. Wong W. L. Bennett G. L. Wrathall J. R. (1991). Increase in nerve growth factor-like immunoreactivity and decrease in choline acetyltransferase following contusive spinal cord injury. Brain Res. 554, 264–271. doi: 10.1016/0006-8993(91)90199-6, PMID: 1933308

[ref4] Beckers M. C. Ernst E. Belcher S. Howe J. Levenson R. Gros P. (1996). A new sodium channel alpha-subunit gene (Scn9a) from Schwann cells maps to the Scn1a, Scn2a, Scn3a cluster of mouse chromosome 2. Genomics 36, 202–205. doi: 10.1006/geno.1996.0447, PMID: 8812438

[ref5] Bennett D. L. Clark A. J. Huang J. Waxman S. G. Dib-Hajj S. D. (2019). The role of voltage-gated sodium channels in pain Signaling. Physiol. Rev. 99, 1079–1151. doi: 10.1152/physrev.00052.2017 30672368

[ref6] Brown A. Ricci M. J. Weaver L. C. (2004). NGF message and protein distribution in the injured rat spinal cord. Exp. Neurol. 188, 115–127. doi: 10.1016/j.expneurol.2004.03.017, PMID: 15191808

[ref7] Brown A. Ricci M. J. Weaver L. C. (2007). NGF mRNA is expressed in the dorsal root ganglia after spinal cord injury in the rat. Exp. Neurol. 205, 283–286. doi: 10.1016/j.expneurol.2007.01.025, PMID: 17335812

[ref8] Burke D. Fullen B. M. Stokes D. Lennon O. (2017). Neuropathic pain prevalence following spinal cord injury: a systematic review and meta-analysis. Eur. J. Pain 21, 29–44. doi: 10.1002/ejp.905, PMID: 27341614

[ref9] Chernov-Rogan T. Li T. Lu G. Verschoof H. Khakh K. Jones S. W.. (2018). Mechanism-specific assay design facilitates the discovery of Nav1.7-selective inhibitors. Proc. Natl. Acad. Sci. U. S. A. 115, E792–e801. doi: 10.1073/pnas.1713701115, PMID: 29311306 PMC5789920

[ref10] Christensen M. D. Hulsebosch C. E. (1997). Spinal cord injury and anti-NGF treatment results in changes in CGRP density and distribution in the dorsal horn in the rat. Exp. Neurol. 147, 463–475. doi: 10.1006/exnr.1997.6608, PMID: 9344570

[ref11] Cox J. J. Reimann F. Nicholas A. K. Thornton G. Roberts E. Springell K.. (2006). An SCN9A channelopathy causes congenital inability to experience pain. Nature 444, 894–898. doi: 10.1038/nature05413, PMID: 17167479 PMC7212082

[ref12] Cummins T. R. Dib-Hajj S. D. Waxman S. G. (2004). Electrophysiological properties of mutant Nav1.7 sodium channels in a painful inherited neuropathy. J. Neurosci. 24, 8232–8236. doi: 10.1523/JNEUROSCI.2695-04.2004, PMID: 15385606 PMC6729696

[ref13] Duan G. Han C. Wang Q. Guo S. Zhang Y. Ying Y.. (2016). A SCN10A SNP biases human pain sensitivity. Mol. Pain 12:174480691666608. doi: 10.1177/1744806916666083, PMID: 27590072 PMC5011395

[ref14] Eijkelkamp N. Linley J. E. Baker M. D. Minett M. S. Cregg R. Werdehausen R.. (2012). Neurological perspectives on voltage-gated sodium channels. Brain 135, 2585–2612. doi: 10.1093/brain/aws225, PMID: 22961543 PMC3437034

[ref15] Eitner A. Hofmann G. O. Schaible H. G. (2017). Mechanisms of osteoarthritic pain. Studies in humans and experimental models. Front. Mol. Neurosci. 10:349. doi: 10.3389/fnmol.2017.00349, PMID: 29163027 PMC5675866

[ref16] Faber C. G. Hoeijmakers J. G. Ahn H. S. Cheng X. Han C. Choi J. S.. (2012). Gain of function Nanu1.7 mutations in idiopathic small fiber neuropathy. Ann. Neurol. 71, 26–39. doi: 10.1002/ana.22485, PMID: 21698661

[ref17] Faber C. G. Lauria G. Merkies I. S. Cheng X. Han C. Ahn H. S.. (2012). Gain-of-function Nav1.8 mutations in painful neuropathy. Proc. Natl. Acad. Sci. U. S. A. 109, 19444–19449. doi: 10.1073/pnas.1216080109, PMID: 23115331 PMC3511073

[ref18] Gwak Y. S. Nam T. S. Paik K. S. Hulsebosch C. E. Leem J. W. (2003). Attenuation of mechanical hyperalgesia following spinal cord injury by administration of antibodies to nerve growth factor in the rat. Neurosci. Lett. 336, 117–120. doi: 10.1016/S0304-3940(02)01251-X, PMID: 12499054

[ref19] Han C. Themistocleous A. C. Estacion M. Dib-Hajj F. B. Blesneac I. Macala L.. (2018). The novel activity of carbamazepine as an activation modulator extends from NaV1.7 mutations to the NaV1.8-S242T Mutant Channel from a patient with painful diabetic neuropathy. Mol. Pharmacol. 94, 1256–1269. doi: 10.1124/mol.118.113076, PMID: 30135145 PMC7501587

[ref20] Hirose M. Kuroda Y. Murata E. (2016). NGF/TrkA Signaling as a therapeutic target for pain. Pain Pract. 16, 175–182. doi: 10.1111/papr.12342, PMID: 26452158

[ref21] Kim D. H. Gutin P. H. Noble L. J. Nathan D. Yu J. S. Nockels R. P. (1996). Treatment with genetically engineered fibroblasts producing NGF or BDNF can accelerate recovery from traumatic spinal cord injury in the adult rat. Neuroreport 7, 2221–2230. doi: 10.1097/00001756-199609020-00033, PMID: 8930993

[ref22] Kim H. Y. Lee H. J. Kim T. L. Kim E. Ham D. Lee J.. (2020). Prevalence and characteristics of neuropathic pain in patients with spinal cord injury referred to a rehabilitation Center. Ann. Rehabil. Med. 44, 438–449. doi: 10.5535/arm.20081, PMID: 33440092 PMC7808793

[ref23] Kozak C. A. Sangameswaran L. (1996). Genetic mapping of the peripheral sodium channel genes, Scn9a and Scn10a, in the mouse. Mamm. Genome 7, 787–788. doi: 10.1007/s003359900235, PMID: 8854872

[ref24] Krenz N. R. Meakin S. O. Krassioukov A. V. Weaver L. C. (1999). Neutralizing intraspinal nerve growth factor blocks autonomic dysreflexia caused by spinal cord injury. J. Neurosci. 19, 7405–7414. doi: 10.1523/JNEUROSCI.19-17-07405.1999, PMID: 10460247 PMC6782501

[ref25] Krenz N. R. Weaver L. C. (2000). Nerve growth factor in glia and inflammatory cells of the injured rat spinal cord. J. Neurochem. 74, 730–739. 10646525 10.1046/j.1471-4159.2000.740730.x

[ref26] Lai J. Hunter J. C. Ossipov M. H. Porreca F. (2000). Blockade of neuropathic pain by antisense targeting of tetrodotoxin-resistant sodium channels in sensory neurons. Methods Enzymol. 314, 201–213. doi: 10.1016/S0076-6879(99)14104-1 10565014

[ref27] Laird J. M. Souslova V. Wood J. N. Cervero F. (2002). Deficits in visceral pain and referred hyperalgesia in Nav1.8 (SNS/PN3)-null mice. J. Neurosci. 22, 8352–8356. doi: 10.1523/JNEUROSCI.22-19-08352.2002, PMID: 12351708 PMC6757795

[ref28] Liu B. W. Zhang J. Hong Y. S. Li N. B. Liu Y. Zhang M.. (2021). NGF-induced Nav1.7 upregulation contributes to chronic post-surgical pain by activating SGK1-dependent Nedd4-2 phosphorylation. Mol. Neurobiol. 58, 964–982. doi: 10.1007/s12035-020-02156-1, PMID: 33063281

[ref29] Ljungberg C. Novikov L. Kellerth J. O. Ebendal T. Wiberg M. (1999). The neurotrophins NGF and NT-3 reduce sensory neuronal loss in adult rat after peripheral nerve lesion. Neurosci. Lett. 262, 29–32. doi: 10.1016/S0304-3940(99)00040-3, PMID: 10076865

[ref30] McDonnell A. Collins S. Ali Z. Iavarone L. Surujbally R. Kirby S.. (2018). Efficacy of the Nav1.7 blocker PF-05089771 in a randomised, placebo-controlled, double-blind clinical study in subjects with painful diabetic peripheral neuropathy. Pain 159, 1465–1476. doi: 10.1097/j.pain.0000000000001227, PMID: 29578944

[ref31] Minett M. S. Nassar M. A. Clark A. K. Passmore G. Dickenson A. H. Wang F.. (2012). Distinct Nav1.7-dependent pain sensations require different sets of sensory and sympathetic neurons. Nat. Commun. 3:791. doi: 10.1038/ncomms1795, PMID: 22531176 PMC3337979

[ref32] Nassar M. A. Stirling L. C. Forlani G. Baker M. D. Matthews E. A. Dickenson A. H.. (2004). Nociceptor-specific gene deletion reveals a major role for Nav1.7 (PN1) in acute and inflammatory pain. Proc. Natl. Acad. Sci. U. S. A. 101, 12706–12711. doi: 10.1073/pnas.0404915101, PMID: 15314237 PMC515119

[ref33] Obata K. Yamanaka H. Dai Y. Mizushima T. Fukuoka T. Tokunaga A.. (2004). Activation of extracellular signal-regulated protein kinase in the dorsal root ganglion following inflammation near the nerve cell body. Neuroscience 126, 1011–1021. doi: 10.1016/j.neuroscience.2004.04.036, PMID: 15207334

[ref34] Peng C. Li L. Zhang M. D. Bengtsson Gonzales C. Parisien M. Belfer I.. (2017). miR-183 cluster scales mechanical pain sensitivity by regulating basal and neuropathic pain genes. Science 356, 1168–1171. doi: 10.1126/science.aam7671, PMID: 28572455

[ref0010] Raitano A. B. Raitano J. R. Hambuch T. M. Sawyers C. L. (1995). The Bcr-Abl leukemia oncogene activates Jun kinase and requires Jun for transformation. Proc. Natl. Acad. Sci. U S A 92, 11746–50. doi: 10.1007/BF01187077, PMID: 8524841 PMC40479

[ref35] Rich K. M. Disch S. P. Eichler M. E. (1989). The influence of regeneration and nerve growth factor on the neuronal cell body reaction to injury. J. Neurocytol. 18, 569–576. doi: 10.1007/BF01187077, PMID: 2614478

[ref36] Sangameswaran L. Delgado S. G. Fish L. M. Koch B. D. Jakeman L. B. Stewart G. R.. (1996). Structure and function of a novel voltage-gated, tetrodotoxin-resistant sodium channel specific to sensory neurons. J. Biol. Chem. 271, 5953–5956. doi: 10.1074/jbc.271.11.5953, PMID: 8626372

[ref37] Sangameswaran L. Fish L. M. Koch B. D. Rabert D. K. Delgado S. G. Ilnicka M.. (1997). A novel tetrodotoxin-sensitive, voltage-gated sodium channel expressed in rat and human dorsal root ganglia. J. Biol. Chem. 272, 14805–14809. doi: 10.1074/jbc.272.23.14805, PMID: 9169448

[ref38] Shiao R. Lee-Kubli C. A. (2018). Neuropathic pain after spinal cord injury: challenges and research perspectives. Neurotherapeutics 15, 635–653. doi: 10.1007/s13311-018-0633-4, PMID: 29736857 PMC6095789

[ref39] Sun L. Fleetwood-Walker S. Mitchell R. Joosten E. A. Cheung C. W. (2020). Prolonged analgesia by spinal cord stimulation following a spinal injury associated with activation of adult neural progenitors. Pain Pract. 20, 859–877. doi: 10.1111/papr.12921, PMID: 32474998

[ref40] Sun L. Xia R. Jiang J. Wen T. Huang Z. Qian R.. (2021). MicroRNA-96 is required to prevent allodynia by repressing voltage-gated sodium channels in spinal cord. Prog. Neurobiol. 202:102024. doi: 10.1016/j.pneurobio.2021.102024, PMID: 33636225

[ref41] Toledo-Aral J. J. Moss B. L. He Z. J. Koszowski A. G. Whisenand T. Levinson S. R.. (1997). Identification of PN1, a predominant voltage-dependent sodium channel expressed principally in peripheral neurons. Proc. Natl. Acad. Sci. U. S. A. 94, 1527–1532. doi: 10.1073/pnas.94.4.1527, PMID: 9037087 PMC19825

[ref42] Werhagen L. Hultling C. Molander C. (2007). The prevalence of neuropathic pain after non-traumatic spinal cord lesion. Spinal Cord 45, 609–615. doi: 10.1038/sj.sc.3102000, PMID: 17160075

[ref43] Widerström-Noga E. (2017). Neuropathic pain and spinal cord injury: phenotypes and pharmacological management. Drugs 77, 967–984. doi: 10.1007/s40265-017-0747-8, PMID: 28451808

[ref44] Xue Y. Kremer M. Muniz Moreno M. D. M. Chidiac C. Lorentz R. Birling M. C.. (2022). The human SCN9A (R185H) point mutation induces pain hypersensitivity and spontaneous pain in mice. Front. Mol. Neurosci. 15:913990. doi: 10.3389/fnmol.2022.913990, PMID: 35769334 PMC9234669

[ref45] Yang Y. Wang Y. Li S. Xu Z. Li H. Ma L.. (2004). Mutations in SCN9A, encoding a sodium channel alpha subunit, in patients with primary erythermalgia. J. Med. Genet. 41, 171–174. doi: 10.1136/jmg.2003.012153, PMID: 14985375 PMC1735695

[ref46] Yu H. Y. Mu D. G. Chen J. Yin W. (2011). Suppressive effects of intrathecal paeoniflorin on bee venom-induced pain-related behaviors and spinal neuronal activation. Pharmacology 88, 159–166. doi: 10.1159/000330456, PMID: 21934352

[ref47] Zain M. Bonin R. P. (2019). Alterations in evoked and spontaneous activity of dorsal horn wide dynamic range neurons in pathological pain: a systematic review and analysis. Pain 160, 2199–2209. doi: 10.1097/j.pain.0000000000001632, PMID: 31149976

