# Supplementary material for: Ectopic expression of Nav1.7 in spinal dorsal horn neurons induced by NGF contributes to neuropathic pain in a mouse spinal cord injury model
Source: Front Mol Neurosci. 2023 Mar 3;16:1091096. doi: 10.3389/fnmol.2023.1091096 (PMC10020601; doi:10.3389/fnmol.2023.1091096)
Supplement: Supplementary file 1 [file Image_1.pdf]

Supplementary information

Supplementary Figures

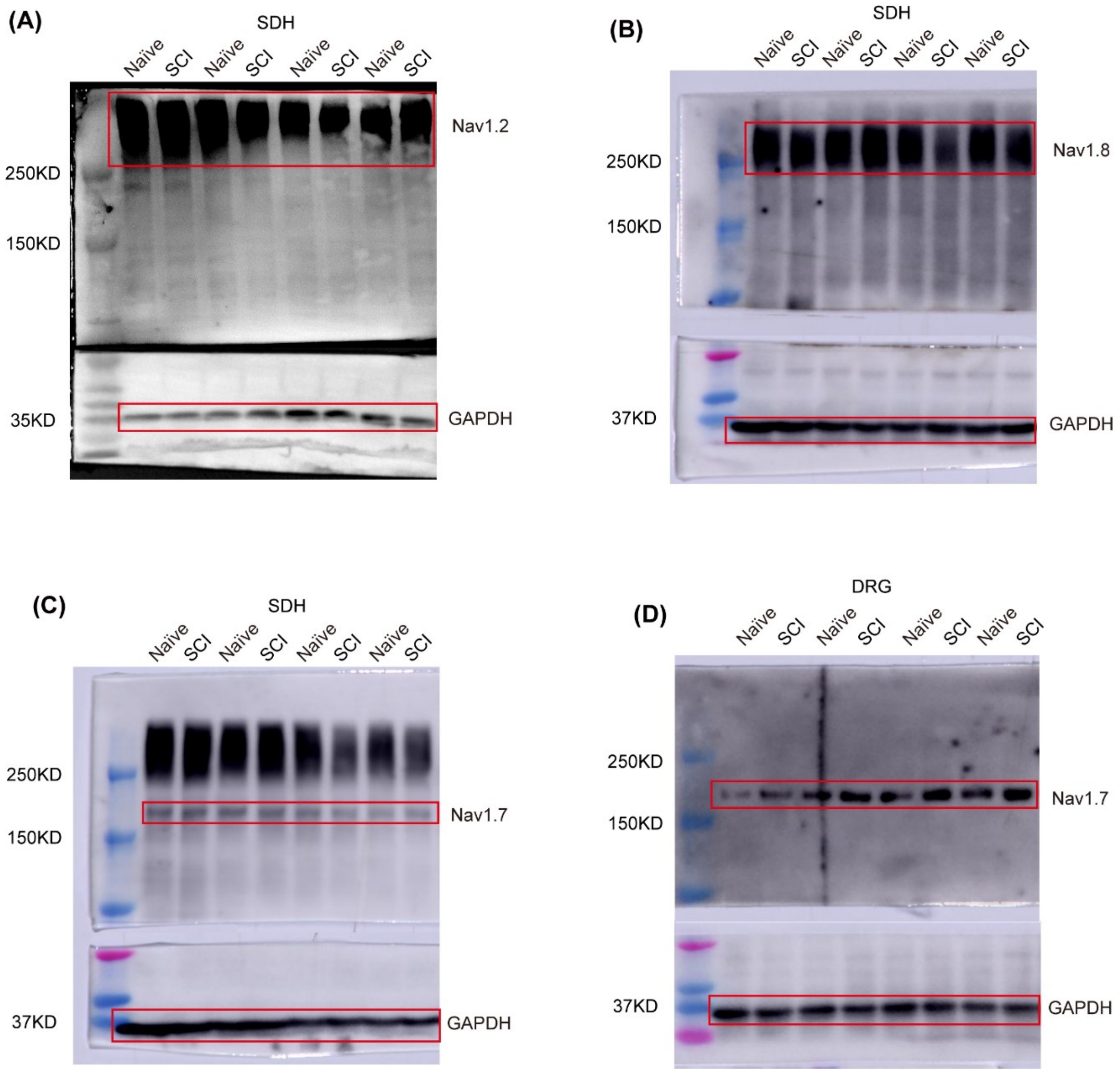

Supplementary Figure 1. The entire image of Western-blot membranes showed in Fig. 2.

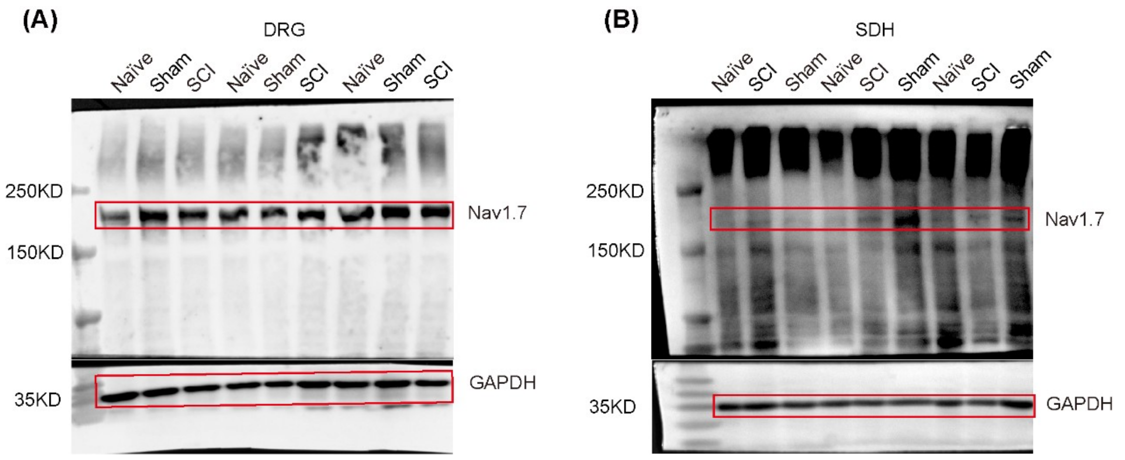

Supplementary Figure 2. The entire image of Western-blot membranes related to Fig. 3.

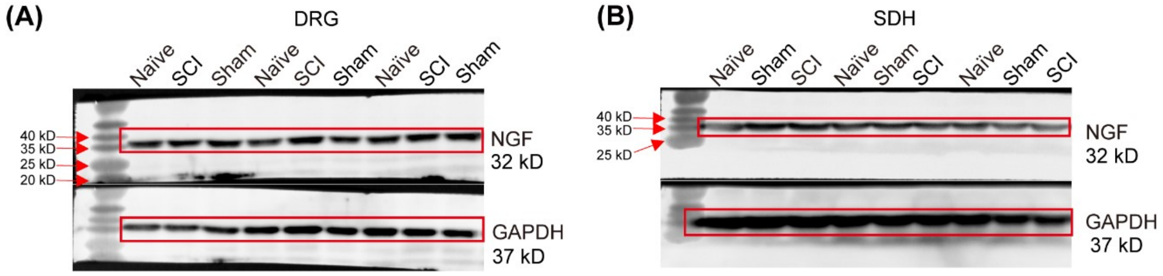

Supplementary Figure 3. The entire image of Western-blot membranes related to Fig. 6.

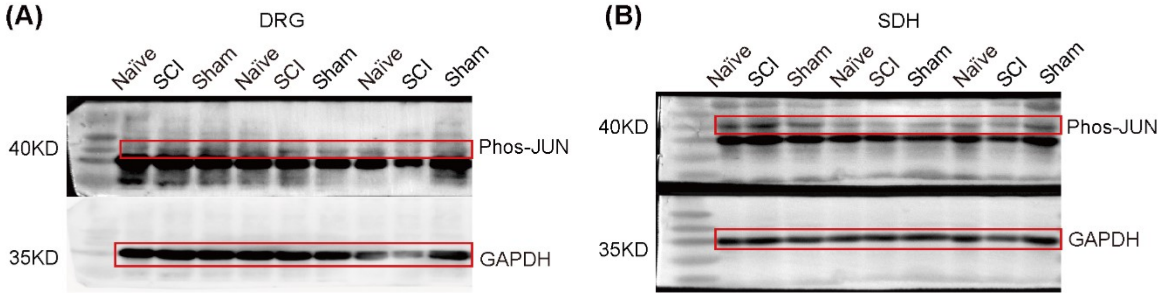

Supplementary Figure 4. The entire image of Western-blot membranes related to Fig. 7.
